# Clinical performance of ASTA SepsiPrep kit in direct bacterial identification and antimicrobial susceptibility test using MicroIDSys Elite and VITEK‐2 system

**DOI:** 10.1002/jcla.23744

**Published:** 2021-05-03

**Authors:** In Young Yoo, Jayho Han, Sung Il Ha, Young Jong Cha, Shin Dong Pil, Yeon‐Joon Park

**Affiliations:** ^1^ Department of Laboratory Medicine College of Medicine Seoul St. Mary's Hospital The Catholic University of Korea Seoul Korea

**Keywords:** AST, direct identification, MALDI‐TOF, positive blood culture, SepsiPrep

## Abstract

**Background:**

Rapid and accurate microbial identification and antimicrobial susceptibility testing (AST) are essential for timely use of appropriate antimicrobial agents for bloodstream infection. To shorten the time for isolating colonies from the positive blood culture, various preparation methods for direct identification using matrix‐assisted laser desorption/ionization time‐of‐flight mass spectrometry (MALDI‐TOF MS) system were developed. Here, we evaluated the SepsiPrep kit (ASTA Corp.) for direct identification of microorganisms and AST from positive blood cultures using MicroIDSys Elite MALDI‐TOF MS system (ASTA Corp.) and VITEK‐2 system (bioMérieux).

**Methods:**

For direct identification, a total of 124 prospective monomicrobial positive blood culture bottles were included. For direct identification, the pellet was prepared by centrifugation and washing twice. For direct AST, the pellet was suspended in 0.45% saline and adjusted to McFarland 0.5. The results from the direct identification and AST using MicroIDSys Elite and VITEK‐2 system were compared to those from the conventional method performed with pure colony subcultured on agar plate.

**Results:**

Compared to the conventional method using pure colony, correct direct identification rate was 96.5% and 98.5% for 57 gram‐positive isolates and 67 gram‐negative isolates, respectively. For direct AST, among the 55 gram‐positive isolates, the categorical agreement (CA) for staphylococci, streptococci, and enterococci was 96.7%, 98.4%, and 94.1%, respectively. For 66 gram‐negative isolates, the CA for *Enterobacterales* and non‐fermentative gram‐negative rods was 99.0% and 96.6%, respectively.

**Conclusions:**

The SepsiPrep kit was easy to use combined with MicroIDSys Elite and VITEK‐2 system and also, the correct identification and AST rate were very high.

## 1 INTRODUCTION

A bloodstream infection is a life‐threatening situation with high mortality rate, approximately 15%–30%.^1^ Rapid, accurate diagnostic tests, and innovative treatments constitute the key for improvement of bloodstream infection outcome.^2^ The conventional method to detect infectious agents from the positive blood cultures usually takes up to several days due to subculturing onto solid agar plates and biochemical tests.

Recently, matrix‐assisted laser desorption/ionization time‐of‐flight mass spectrometry (MALDI‐TOF MS), which can identify the microorganisms from the colonies within minutes, has proven over the years to be a rapid and accurate method for identification of microorganisms.^3^ To circumvent the time required for colony formation, several sample preparation procedures for blood culture pellet prior to MALDI‐TOF MS analysis have been studied for identification of microorganisms to shorten the diagnostic procedure: Various procedures which include 1) using chemical reagents such as saponin and ammonium chloride solution and 2) using centrifugation for separation of microorganisms.^4^, ^5^, ^6^, ^7^, ^8^ Also, commercially kit such the Bruker MALDI Sepsityper Kit (Bruker Daltonics) became available for microorganism identification.^9^ However, antimicrobial susceptibility testing (AST) is essential for selecting appropriate antibiotics. In our earlier study, we developed a sample preparation method which can be used for not only for antimicrobial susceptibility test but also for AST.^8^, ^10^

Very recently, the SepsiPrep kit (ASTA Corp.) which can be used for direct identification of microorganisms from positive blood culture was introduced. Therefore, we evaluated the SepsiPrep kit for direct identification of microorganisms and AST from positive blood cultures using MicroIDSys Elite system (ASTA Corp.,) and VITEK‐2 system (bioMérieux), respectively.

## 2 MATERIALS AND METHODS

### 2.1 Study isolates

We prospectively evaluated 124 blood cultures flagged as positive for bacterial growth from August to September 2020. Blood cultures were collected in BACTEC Plus aerobic/F and Anaerobic/F bottles (Becton Dickinson) and incubated in the BACTEC FX blood culture system (Becton Dickinson). Direct identification and AST were performed in parallel to the conventional method in all enrolled blood cultures. Blood cultures with multiple organisms by Gram stain were excluded. This study was approved by the Institutional Review Board of Seoul St. Mary's Hospital (IRB‐KC18DND10866).

### 2.2 Conventional identification and AST

When automated blood culture system showed positive signal, an aliquot from positive blood cultures was subjected to Gram staining and then subcultured onto the following agar plates: sheep blood agar (Asan Pharmaceutical Co., Ltd.) and MacConkey agar (Asan Pharmaceutical. Co., Ltd.). After overnight incubation, a pure colony from the agar plate was used for MALDI‐TOF MS analysis with MicroIDSys system and AST by VITEK‐2 system.

For MALDI‐TOF MS analysis, colonies were smeared onto the target plate, 1.5 µL of 70% formic acid (ASTA Corp.) and 1.5 µL of a α‐cyano‐4‐hydroxycinnamic acid matrix solution (ASTA Corp.) were added for analysis with the MicroIDSys system. Acquired protein spectrum was compared with the reference library provided by the manufacturer (MicroIDSys CoreDB v1.27), and the score ≥140 was considered acceptable according to the manufacturer's recommendation.

The AST of the isolates was performed VITEK‐2 system (AST P601 card for staphylococci, AST‐ST01 card for streptococci, AST‐P600 card for enterococci, AST‐N224 card for Enterobacteriaceae, and AST‐N225 cards for non‐fermentative gram‐negative rods) (bioMérieux). For AST, a McFarland 0.5 standard suspension was prepared using 0.45% saline with the Densichek VITEK colorimeter. The minimum inhibitory concentration results were resolved into the three clinical categories (susceptible, intermediate, and resistant) according to the Clinical and Laboratory Standards Institute's (CLSI) M100 document.^11^ However, for several agents and/or organism groups (eg, tigecycline for any bacteria, fusidic acid and habekacin for *Staphylococcus* spp., and moxifloxacin for *Streptococcus* spp. viridans group or *Streptococcus* spp. β‐hemolytic Group) where there are no CLSI breakpoints, the breakpoints provided by manufacturer was applied.

### 2.3 Direct identification and AST

When a blood culture was flagged positive by the BACTEC FX blood culture system (Becton Dickinson) indicating bacterial growth, and a Gram stain confirmed the presence of the gram‐positive or gram‐negative bacteria. For cases showing single morphotype by Gram stain, direct bacterial identification was performed using SepsiPrep kit (ASTA Corp.). Briefly, 1 ml of blood culture was transferred to a lysis tube. After vortex for 2 minutes, the tube was centrifuged at 13,000 *g* for 2 minutes, and supernatant was discarded. Then, the pellet was washed twice with 1 ml of wash solution by vortexing and centrifugation (2 min, 13,000 *g*). The supernatant was discarded, and the pellet was used for MALDI‐TOF MS analysis. For AST, the pellet was suspended in 0.45% saline and adjusted to McFarland 0.5.

The MALDI‐TOF MS analysis and AST are performed in the same way as colony with conventional method. For the AST, the unidentified isolates from direct identification were excluded because VITEK cards were chosen according to the identification obtained by MALDI‐TOF. The identification was introduced into the VITEK 2 expert system to allow it to choose the correct interpretive criteria.

### 2.4 Data analysis

For identification, samples determined to be incorrectly identified were those with invalid identification, and samples with inconsistent results between the direct identification and conventional identification methods. The correct identification rate was calculated as (correctly identified samples/total tested samples) × 100.

Comparison of AST between the direct and conventional method was expressed in terms of categorical agreement (CA), very major error (VME, falsely susceptible), major error (ME, falsely resistant), or minor error (mE, all other errors) according to the CLSI M23Ed5 document.^12^

## 3 RESULTS

### 3.1 Identification

One hundred and twenty‐four monomicrobial positive blood culture were enrolled in this study, with 57 gram‐positive isolates and 67 gram‐negative isolates. Compared to the conventional method, correct identification rates were 96.5% and 98.5% for the 57 gram‐positive and the 67 gram‐negative isolates, respectively (Table 1). A total of three isolates showed “invalid identification”; *Streptococcus mitis/oralis* (*n* = 1), *Enterococcus faecium* (*n* = 1), and *Klebsiella oxytoca* (*n* = 1).

**TABLE 1:** Proportion of correct identification rate among the 57 gram‐positive isolates and 67 gram‐negative isolates

Microorganisms	No. of isolates	Correct identification	Microorganisms	No. of isolates	Correct identification
Gram‐positive isolates			Gram‐negative isolates		
*Staphylococcus* aureus	12	12	*Escherichia coli*	32	32
*Staphylococcus epidermidis*	12	12	*Enterobacter cloacae*	1	1
*Staphylococcus capitis*	4	4	*Klebsiella aerogenes*	2	2
*Staphylococcus haemolyticus*	1	1	*Klebsiella oxytoca*	6	5
*Staphylococcus hominis*	1	1	*Klebsiella pneumoniae*	18	18
*Streptococcus mitis/oralis*	5	4	*Serratia marcescens*	1	1
*Streptococcus agalactiae*	1	1	*Acinetobacter baumannii*	1	1
*Enterococcus faecalis*	15	15	*Pseudomonas aeruginosa*	4	4
*Enterococcus faecium*	6	5	*Pseudomonas putida*	2	2
Total	57	55 (96.5%)	Total	67	66 (98.5%)

### 3.2 Antimicrobial susceptibility testing

Among the 124 enrolled blood cultures, 121 which were identified by direct identification were selected for direct AST.

The AST results for 30 *Staphylococcus* spp., 20 *Enterococcus* spp., and 5 *Streptococcus* spp. are presented in Table 2. For 55 gram‐positive isolates, 510, 220, and 64 bacteria/antimicrobial agent combinations were analyzed for staphylococci, enterococci, and streptococci, respectively. The CA for staphylococci, enterococci, and streptococci was 96.7%, 94.1%, and 98.4%, respectively. For 30 staphylococci, among the 17 antimicrobial agents tested, the CA was ≥90% for all of them except teicoplanin with which mE rate was 13.3%. Among the 510 bacteria/antimicrobial agent combinations, the VMEs, MEs, and mEs were 0.8% (4/510), 0.2% (1/510), and 2.4% (12/510), respectively. The VMEs were observed for tigecycline (2 isolates) and trimethoprim‐sulfamethoxazole (2 isolates). For 20 enterococci, among the 11 antimicrobial agents tested, the CA was ≥90% for all of them except erythromycin (85.5%) and teicoplanin (75.5%). While all the errors found with erythromycin were mEs, those for teicoplanin were VMEs. Among the 220 bacteria/antimicrobial agent combinations, the VMEs, MEs, and mEs were 3.6% (8/220), 0.0% (0/220), and 2.3% (5/220), respectively. The VMEs were found with teicoplanin (5 isolates), benzylpenicillin (2 isolates), and high‐level gentamicin (1 isolate). For five *Streptococcus* spp., among the 13 antimicrobial agents tested, the CA was all 100% only except moxifloxacin (75%) which was 1 minor error among the 64 isolate/antimicrobial agent combinations.

**TABLE 2:** The accuracy of antimicrobial susceptibility test results obtained using the blood culture pellet versus conventional method VITEK‐2 system among 55 gram‐positive isolates

Microorganisms	Antimicrobial agent	CA (No. (%))	No. of strains with:		
			Very major error	Major error	Minor error
*Staphylococcus* spp. (*n* = 30)	Benzylpenicillin	30 (100)			
	Oxacillin	30 (100)			
	Gentamicin	27 (90.0)			3
	Hebekacin	30 (100)			
	Ciprofloxacin	29 (96.7)			1
	Erythromycin	30 (100)			
	Telithromycin	28 (93.3)			2
	Clindamycin	29 (96.7)		1	
	Linezolid	30 (100)			
	Teicoplanin	26 (86.7)			4
	Vancomycin	30 (100)			
	Tetracycline	28 (93.3)			2
	Tigecycline	28 (93.3)	2		
	Nitrofurantoin	30 (100)			
	Fusidic acid	30 (100)			
	Rifampin	30 (100)			
	Trimethoprim/Sulfamethoxazole	28 (93.3)	2		
	Total	493 (96.7)	4	1	12
*Enterococcus* spp. (*n* = 20)	Benzylpenicillin	18 (90.0)	2		
	Ampicillin	20 (100)			
	Ampicillin/Sulbactam	20 (100)			
	Gentamicin high level	19 (95.5)	1		
	Streptomycin high level	20 (100)			
	Erythromycin	17 (85.5)			3
	Quinupristin/Dalfopristin	19 (95.5)			1
	Linezolid	20 (100)			
	Teicoplanin	15 (75.5)	5		
	Vancomycin	20 (100)			
	Tigecycline	19 (95.5)			1
	Total	207 (94.1)	8	0	5
*Streptococcus* spp. (*n* = 5)	Benzylpenicillin	5 (100)			
	Ampicillin	5 (100)			
	Cefotaxime	5 (100)			
	Ceftriaxone	5 (100)			
	Levofloxacin	5 (100)			
	Moxifloxacin	3 (75.0)			1
	Erythromycin	5 (100)			
	Clindamycin	5 (100)			
	Linezolid	5 (100)			
	Vancomycin	5 (100)			
	Tetracycline	5 (100)			
	Tigecycline	5 (100)			
	Chloramphenicol	5 (100)			
	Total	63 (98.4)	0	0	1

Abbreviation: CA, Categorical agreement.

For 66 gram‐negative isolates, 1003 and 89 bacteria/antimicrobial agent combinations were analyzed for *Enterobacterales* and non‐fermentative gram‐negative rods, respectively. The CA for *Enterobacterales* and non‐fermentative gram‐negative rods was 99.0% and 96.6%, respectively. For *Enterobacterales*, among the 1003 bacteria/antimicrobial agent combinations, the VMEs, MEs, and mEs were 0.1% (1/1003), 0.0% (0/1003), and 0.9% (9/1003), respectively. The only one VME was observed for trimethoprim/sulfamethoxazole, and mEs were most frequently found in ciprofloxacin (4 isolates) and was followed by piperacillin/tazobactam (2 isolates) (Table 3).

**TABLE 3:** The accuracy of antimicrobial susceptibility test results obtained using the blood culture pellet versus conventional method VITEK‐2 system among 66 gram‐negative isolates

Microorganisms	Antimicrobial agent	CA (No. (%))	No. of strains with:		
			Very major error	Major error	Minor error
*Enterobacterales* (*n* = 59)	Ampicillin	59 (100)			
	Amoxicillin/Clavulanate	58 (98.3)			1
	Piperacillin/Tazobactam	57 (96.7)			2
	Cefazolin	59 (100)			
	Cefoxitin	58 (98.3)			1
	Cefotaxime	59 (100)			
	Ceftazidime	58 (98.3)			1
	Cefepime	59 (100)			
	Aztreonam	59 (100)			
	Ertapenem	59 (100)			
	Imipenem	59 (100)			
	Amikacin	59 (100)			
	Gentamicin	59 (100)			
	Ciprofloxacin	55 (93.3)			4
	Tigecycline	59 (100)			
	Trimethoprim/Sulfamethoxazole	58 (98.3)	1		
	Meropenem	59 (100)			
	Total	993 (99.0)	1	0	9
Non‐fermentative Gram‐negative rods (*n* = 7)	Ampicillin/Sulbactam	1 (100)			
	Ticarcillin/Clavulanate	6 (85.7)			1
	Piperacillin	7 (100)			
	Piperacillin/Tazobactam	7 (100)			
	Cefotaxime	3 (100)			
	Ceftazidime	7 (100)			
	Cefepime	7 (100)			
	Imipenem	7 (100)			
	Meropenem	7 (100)			
	Amikacin	7 (100)			
	Gentamicin	7 (100)			
	Ciprofloxacin	7 (100)			
	Minocycline	2 (66.7)			1
	Tigecycline	2 (66.7)			1
	Trimethoprim/Sulfamethoxazole	3 (100)			
	Aztreonam	6 (100)			
	Total	86 (96.6)	0	0	3

Abbreviation: CA, Categorical agreement.

For non‐fermentative gram‐negative rods, among the 89 bacteria/antimicrobial agent combinations, VME and ME were not found and only three mEs were found with each of the following drugs; ticarcillin/clavulanate, minocycline, and tigecycline (Table 3).

The bacteria/antimicrobial agent combinations that did not agree with conventional method are listed in Table 4.

**TABLE 4:** Discrepancies of antimicrobial susceptibility testing results between the direct method and the conventional method

Microorganism	No. (%) of strains with:		
	Very major error (*n* = 13)	Major error (*n* = 1)	Minor error (*n* = 30)
*Staphylococcus aureus*	Tigecycline (2)		Telithromycin (1)
*Staphylococcus capitis*			Gentamicin (1) Teicoplanin (1)
*Staphylococcus epidermidis*	Trimethoprim/Sulfamethoxazole (2)	Clindamycin (1)	Gentamicin (1) Ciprofloxacin (1) Telithromycin (1) Teicoplanin (3) Tetracycline (2)
*Staphylococcus hominis*			Gentamicin (1)
*Streptococcus mitis/oralis*			Moxifloxacin (1)
*Enterococcus faecalis*	Benzylpenicillin (2)		Erythromycin (2) Tigecycline (1)
*Enterococcus faecium*	Gentamicin High‐Level (1) Teicoplanin (5)		Erythromycin (1) Quinupristin/Dalfopristin (1)
*Escherichia coli*	Trimethoprim/Sulfamethoxazole (1)		Amoxicillin/Clavulanate (1) Piperacillin/Tazobactam (2) Ceftazidime (1) Ciprofloxacin (3)
*Klebsiella pneumoniae*			Cefoxitin (1) Ciprofloxacin (1)
*Pseudomonas aeruginosa*			Ticarcillin/Clavulanate (1)
*Pseudomonas putida*			Minocycline (1) Tigecycline (1)

## 4 DISCUSSION

Rapid and accurate microbial identification and AST are critical for timely use of appropriate antimicrobial agents for bloodstream infection.^2^ Therefore, to shorten the turnaround time from the positive signal of the blood culture bottle to the identification of the microorganism, we and several researchers have developed sample preparation method using saponin/filtration method,^8^ lysis/filtration,^13^ or lysis/extraction.^10^ Although it is difficult to compare the accuracy of various sample preparation methods, in general, the correct identification rate for gram‐positive bacteria was lower (63.3% to 92.6%) than gram‐negative bacteria (82.8% to 97.7%).^8^, ^10^, ^14^ In this study, though the number of enrolled isolates was small and the species were not diverse, the correct identification rate to the species level among the 124 isolates was 97.6% (121/124). Considering that the correct identification rate for gram‐positive isolates was much lower (73.9%, 108/149) in our previous study^8^ where we used saponin and nylon mesh filter, and the discrepancy was mainly due to the low correction rate among the *S. epidermidis* (78.3%, 18/23), *S. capitis* (62.5%, 5/8), *S. mitis*/*oralis* (37.5%, 3/8), and *E. faecium* (91.3%, 21/23) among the 149 gram‐positive isolates tested, the performance of the ASTA SepsiPrep kit was excellent and was comparable to the lysis/filtration method^13^ which is more cumbersome.

For direct AST, several studies reported the CA rate of around 92.3% to 97.8% for gram‐positive isolates^8^, ^13^, ^14^, ^15^ but it is difficult to compare because there is a difference not only in sample preparation method but also in antimicrobial agents included in each study. For instance, in our study, the major source of error in gram‐positive isolates was teicoplanin (for *Staphylococcus* spp. and *Enterococcus* spp.) and erythromycin (for *Enterococcus* spp.) and moxifloxacin (for *Streptococcus* spp.). Pan *et al*.^15^ reported that the CA was <90% in moxifloxacin, levofloxacin, clindamycin for *Staphylococcus* spp., and ciprofloxacin for *Enterococcus* spp., but in their study, either teicoplanin or high‐level gentamicin was not included. For antimicrobial agents with high error rates, AST results should be confirmed. Especially for teicoplanin, considering high proportion of VanA‐type vancomycin‐resistant isolates conferring inducible high‐level resistance to both vancomycin and teicoplanin, a confirmatory test is needed.^16^

Regarding gram‐negative isolates, the CA for both *Enterobacterales* and non‐fermentative gram‐negative rods was very high (99.0% and 96.6%, respectively). This is comparable or higher than that obtained in other previous studies which conducted with VITEK‐2 system for AST using blood culture pellet.^8^, ^13^, ^14^, ^15^ Recently, Pan *et al*. reported that CA of antimicrobials against gram‐negative isolates was 96.9% (97.9% for *Enterobacterales* and 93.2% for non‐fermenter, respectively).^15^ In our study, only one VME was found with trimethoprim/sulfamethoxazole and this is in line with Pan *et al*. in that VME was found only with trimethoprim/sulfamethoxazole among the *Enterobacterales* and it was also major source of VME in a study by Romero‐Gomez *et al*.^14^

For non‐fermentative gram‐negative rods, the CA for each antimicrobial agent was above 90% except for ticarcillin/clavulanate, minocycline, and tigecycline. Meanwhile, Romero‐Gomez *et al*.reported that VMEs in non‐fermenter mainly occurred with piperacillin‐tazobactam, ticarcillin, and trimethoprim‐sulfamethoxazole among the 26 non‐fermentative gram‐negative rods but high agreement with minocycline and tigecycline.^14^

Our study has a limitation in that relatively small number of isolates were analyzed, especially for non‐fermentative gram‐negative rods. Nevertheless, our study demonstrates the effectiveness of the blood culture pellet prepared with SepsiPrep kit for direct AST as well as rapid and accurate identification. The ASTA SepsiPrep kit was very easy to use because the lysis buffer is freeze‐dried and contained in one tube and only two washing steps are needed. Combining use of MicroIDSys Elite system and VITEK‐2 system with blood culture pellet do provide reliable identification and AST results in same day that the blood culture bottles flagged positive.

## CONFLICT OF INTEREST

No potential conflicts of interest relevant to this paper were reported.

## AUTHOR CONTRIBUTIONS

YJ Park designed the study. YJ Park and IY Yoo analyzed the data and wrote the manuscript. YJ Cha collected the samples. DP Shin, SI Ha, and JH Han participated in experiments. YJ Park supervised the study design and reviewed the manuscript. All authors read and approved the final manuscript.

## Data Availability

The data that support the findings of this study are available from the corresponding author upon reasonable request.
